# The profile of offenders accused of rape referred to the Observation Ward of the Free State Psychiatric Complex, Bloemfontein, South Africa, 2011–2015

**DOI:** 10.4102/sajpsychiatry.v28i0.1807

**Published:** 2022-05-31

**Authors:** Laurisa van Zyl, Nathaniel L. Mosotho, Leilani Cronjé, Mia Vosloo, Leandré Odendaal, Fritz Odendaal, Sarel Kruger, Gina Joubert

**Affiliations:** 1 Department of Psychiatry, Faculty of Health Sciences, University of the Free State, Bloemfontein, South Africa; 2 Department of Biostatistics, Faculty of Health Sciences, University of the Free State, Bloemfontein, South Africa

**Keywords:** sociodemographics, mental illness, clinical, accused offenders, rape, forensic, assessment, profile, outcomes

## Abstract

**Background:**

Sexual assault in South Africa has reached alarming proportions. Mental illness amongst offenders accused of rape is one of the complicating factors.

**Aim:**

To describe the sociodemographic, clinical and forensic profile of alleged offenders accused of rape referred to a South African tertiary public psychiatric hospital for forensic evaluation, as well as describe the profile of the reported victims.

**Setting:**

Free State Psychiatric Complex (FSPC) Observation Ward, Bloemfontein, South Africa.

**Methods:**

This cross-sectional retrospective study compiled a sociodemographic, clinical and forensic profile of offenders accused of rape referred to the FSPC for forensic assessment between 2011 and 2015. The study population comprised 159 alleged offenders. Sociodemographic data, mental status, clinical diagnoses, comorbid substance use and forensic outcomes were collected from clinical files.

**Results:**

All accused offenders were male with a median age of 30 years. About half obtained primary school level education. Unemployment was common (37.3%) with 40% receiving government grants. Most accused offenders were single (78.0%). Clinically, schizophrenia spectrum and other psychotic disorders were the most common diagnoses (30.8%), followed by intellectual disability (16.3%). Almost a third of the accused offenders did not suffer from any mental illness. Alcohol (20.1%) and cannabis use disorders (12.6%) were dominant comorbidities. After a 30-day evaluation period, 53.2% of alleged offenders were found competent to stand trial. The verdict of criminal responsibility was made for 43.7% of this sample.

**Conclusion:**

This study contributes data on sociodemographics, psychiatric morbidities and comorbidities and forensic findings amongst alleged offenders of rape referred for forensic assessment.

## Introduction

The 2018 World Health Organization’s Burden of Disease report indicated that the lifetime prevalence of physical and sexual intimate partner violence and non-partner sexual violence amongst women aged 15 years and older was 30%.^1^ In South Africa, an average of 116 rape cases are reported to the South African Police Service (SAPS) daily.^2^ Despite the underreporting of sex-related crimes, the statistics remain high, suggesting that reported cases are only the tip of the iceberg.^2^ The presence of mental disorders amongst alleged offenders of rape may be one of the complicating factors.

*The Criminal Procedure Act* No. 51 of 1977’s sections 77, 78 and 79 cover the relationship between mental health and crime in South Africa.^3^ This Act mainly addresses three legal constructs, namely, competency to stand trial, criminal responsibility and the expert report on the mental status of the accused. Section 77 deals with the capacity of the accused to stand trial. In other words, this section interrogates whether alleged offenders understand and follow court or legal proceedings appropriately. It has to do with their ability to comprehend the charges brought against them and their capacity to provide proper instructions to their legal teams. Section 78 inquires about the mental status of the alleged offender during the commission of the crime. The question is whether he or she was able to differentiate between right and wrong and exercise the power of resistance to act at that particular junction. Section 79 orders forensic mental health professionals to provide a comprehensive report to the court about the mental status of the accused as well as recommendations on fitness to stand trial and criminal responsibility.^3^

Many studies on the link between mental health and sexual offences, including rape, have been conducted, both locally and globally, reporting various results. At a tertiary public psychiatric hospital in South Africa, Strydom et al.^4^ reported that 35% of their study population of mentally ill persons had committed rape or sex-related crimes, with patients suffering from schizophrenia dominating this sample. However, this differs from a study conducted by Vinkers et al.^5^ in the Netherlands, where rape was least prevalent compared to other crimes, such as battery and manslaughter, amongst patients suffering from psychotic disorders. Additionally, these researchers reported that individuals diagnosed with developmental disorders were most likely to commit sex-related offences. A systematic review by Chua et al.^6^ showed that a high proportion of first-time older offenders accused of rape presented with neurocognitive disorders. As far as substance use or substance use disorders are concerned, researchers worldwide express varying opinions regarding the subject. For example, Tiihonen et al.^7^ emphasised that the use of alcohol had no strong relationship with sexual crimes such as rape. Other researchers, however, reported a strong relationship between substance use and substance use disorders and sexual assault.^8,9,10,11,12^

There is a lacuna of literature regarding general sociodemographic, clinical and forensic characteristics of offenders of rape and sexual crimes in South Africa. Previous studies revealed that offenders of rape referred for forensic evaluation were predominantly single men with an average age of 30 years.^4,13,14^ It also seemed that a lower educational level might be considered a factor as far as the crime of rape is concerned.^5^

### Aim

It is against this background that the researchers decided to study the sociodemographic, clinical and forensic profile of alleged offenders accused of rape (as defined by the *Sexual Offences Act* 32 of 2007)^15^ referred to a South African tertiary public psychiatric hospital for forensic evaluation. This study further aimed to determine the outcome of the multi-professional team’s assessment on competency to stand trial and criminal responsibility amongst these alleged offenders, as well as describe the profile of the reported victims.

## Methods

### Study design and setting

This was a cross-sectional retrospective study. The research was conducted at the Free State Psychiatric Complex (FSPC) Observation Ward, Bloemfontein, South Africa.

### Study population

The study population comprised all alleged offenders accused of rape referred for forensic evaluation between 01 January 2011 and 31 December 2015. There is an estimated 4-month waiting period between court orders for a 30-day observation and the actual admission to the FSPC’s Observation Ward. The researchers employed the consecutive sampling method to include all available study subjects, thus ensuring a better representation of the target population and use of clinical information.^16,17^ This method also limited bias as every potentially suitable participant over a substantial period (5 years) could be included in the study.

### Data collection

Researchers used the forensic examination register to identify alleged offenders of rape. All clinical records were retrieved from the clinical records cabinet and archives of FSPC. File numbers were recorded on data forms, and case study numbers were assigned for the purpose of recovering files, if necessary. Information on alleged offenders was captured on a data collection form, which included sociodemographic data, mental status, clinical diagnoses and comorbid substance use disorders, victim details and forensic referral sections (Sections 77, 78 and 79). The findings of the multi-professional team on these sections following a 30-day forensic assessment period were also recorded.

The researchers conducted a pilot study on the first 10 clinical records from 2011 in order to investigate the practical feasibility of this study. These clinical records were included in the main study, and the researchers effected the following change to the data collection form: an additional category ‘mental deficiency’, currently known as ‘intellectual disability’ in the Diagnostic and Statistical Manual of Mental Disorders, fifth edition.^18^

### Data analysis

Quantitative descriptive data analysis was performed by the Department of Biostatistics, Faculty of Health Sciences, University of the Free State. The results were summarised by frequencies and percentages (categorical variables) and medians and ranges (numerical variables).

### Ethical considerations

The protocol was approved by the Health Sciences Research Ethics Committee, University of the Free State, South Africa [HSREC-S 35/2016]. Written permission to conduct the study was obtained from the Free State Department of Health and the FSPC. As the clinical files were used, no informed consent was required in this study. The researchers handled all the information obtained from clinical files in a confidential manner.

## Results

The total number of alleged offenders accused of rape referred for forensic evaluation at FSPC between 01 January 2011 and 31 December 2015 was 159 (out of 674 observati). The sociodemographic characteristics of the alleged offenders of rape are displayed in Table 1.

**TABLE 1:** Sociodemographic characteristics of offenders accused of rape (*n* = 159).

Variable	*n*	%
**Age groups (years)**		
10–19	21	13.2
20–29	52	32.7
30–39	46	28.9
40–49	24	15.1
50–59	12	7.6
60–69	3	1.9
70–79	1	0.6
**Gender**		
Male	159	100.0
Female	0	0
**Level of education**		
None	16	10.1
Primary	75	47.2
Secondary	67	42.1
Tertiary	1	0.6
**Employment status (*n* = 158)**		
Unemployed	59	37.3
Employed, informal sector	29	18.4
Employed, formal sector	1	0.6
Disability or pensioner	63	39.9
Scholar or student	6	3.8
**Marital status**		
Single	124	78.0
Divorced	1	0.6
Married	6	3.8
Widowed	3	1.9
Other	25	15.7

All the study subjects were males aged 14–76 years with a median age of 30 years. Almost 75% of this sample was younger than 40 years of age. Just under 50% of these accused had obtained only a primary school level of education, whilst < 1% achieved a higher education qualification. Most of the accused were single. There was a notable unemployment rate amongst the offenders accused of rape, and more than 30% were on social welfare or disability grants. The clinical diagnoses are presented in Table 2.

**TABLE 2:** Clinical diagnoses amongst alleged rape offenders (*n* = 159).

Diagnosis	*n*	%
**Diagnostic morbidity**		
No mental illness	45	28.3
Schizophrenia spectrum and other psychotic disorders	49	30.8
Bipolar mood disorder	4	2.5
Substance use disorder	6	3.8
Major neurocognitive disorder	4	2.5
Personality change due to general medical condition	1	0.6
Anti-social personality disorder	2	1.3
Borderline intellectual functioning	14	8.8
Intellectual disability	26	16.3
**Epilepsy and related disorders**	8	5.0
**Diagnostic comorbidity (*n* = 114)†**		
No comorbidity	39	24.5
Alcohol use disorder	32	20.1
Cannabis use disorder	20	12.6
Inhalant use disorder	3	1.9
Other single substance use disorder	4	2.5
Multiple substance use disorders	16	10.1

† , This is applicable to 114 accused offenders, as comorbidity was only recorded if there was a primary clinical diagnosis.

A large portion of the offenders accused of rape were diagnosed with schizophrenia spectrum and other psychotic disorders (30.8%), followed by those who were diagnosed with intellectual disability (16.3%). Furthermore, some of the alleged offenders presented with comorbid clinical diagnoses. These diagnostic comorbidities were mainly current alcohol use disorder (20.1%) and cannabis use disorder (12.6%). It is important to note that some accused offenders had multiple substance use disorders (10.1%). The multi-professional team findings after a 30-day forensic assessment period are depicted in Table 3. After a 30-day forensic assessment period at the FSPC Observation Ward, the multi-professional panel found a relatively high percentage of the accused offenders of rape to be criminally responsible (43.7%), whilst 53.2% were found to be competent to stand trial. The majority (39.9%) of accused offenders that were competent to stand trial were also declared criminally responsible for their crimes. Fitness to stand trial and criminal responsibility were also found to be highly influenced by the presence of mental illness or intellectual disability. Of the alleged offenders with intellectual disability, 70.0% were not considered fit to stand trial, compared to 62.6% of those with mental illness and 2.2% of those with no mental illness. Of the alleged offenders with intellectual disability, 51.3% were considered not criminally responsible, compared to 44.6% and 4.4% of patients with mental illness and those without mental illness, respectively.

**TABLE 3:** Criminal responsibility and competency to stand trial (*n* = 159).

Variable	Competent to stand trial		Not competent to stand trial		Total	
	*n*	%	*n*	%	*n*	%
Criminally responsible	63	39.9	6	3.8	69	43.7
Criminally not responsible	1	0.6	54	34.2	55	34.8
Diminished criminal responsibility	20	12.7	14	8.9	34	21.5

**Total**	**84**	**53.2**	**74**	**46.8**	**-**	**-**

The majority of offenders accused of rape (88.2%) had only one victim, 5.7% had two, 3.8% three and 2.5% had four victims. Victims were mainly female (89.1%) and in the age groups 0–9 years (34.3%) and 10–19 years (32.2%), respectively. The perpetrator was mainly an acquaintance of the victim (47.3%) or a stranger (30.2%). The median age of victims of intellectually disabled offenders accused of rape was lower than the median age of victims violated by mentally ill or mentally healthy offenders accused of rape (9, 12 and 15.5 years, respectively). Intellectually disabled offenders accused of rape had the highest percentage of male victims (17.8%) compared to 7.7% of mentally ill and 10.7% of mentally healthy offenders accused of rape. The group of alleged offenders with intellectual disability also had the highest percentage of perpetrators who mostly violated acquaintances (56.8%).

## Discussion

The results of this study show that all the alleged offenders accused of rape were males. Valença et al.^12^ reported a similar tendency in Rio de Janeiro, Brazil. Similarly, a study conducted at FSPC by Strydom et al.,^4^ reported that 96% of their study population were males. The majority of our study subjects were aged between 20 and 30 years, with a median age of 30 years. This finding confirms what was reported by Barrett et al.^13^ and Marais et al.^14^ in studies also performed at FSPC.

As far as education is concerned, it was found that almost half (47.2%) of the alleged offenders only completed primary level education. This was followed by those who had received a secondary level education (42.1%). This finding is in contrast with what was noted in the United States of America, in which 81.4% of their study population had obtained secondary or higher levels of education.^10^ There was a high rate of unemployment (37.3%) amongst the study subjects, and a notable portion were living on government disability or social grants. The high unemployment rate is higher than the 27.6% unemployment rate reported by Statistics South Africa^19^ in the general population of South Africans who are fit to work.

This study revealed that 28.3% of the accused were not suffering from any mental disorders. This finding differs from a Brazilian report in which Valença et al.^12^ found that all offenders accused of sexual crimes were mentally healthy after forensic assessments. Schizophrenia spectrum and other psychotic disorders and intellectual disability were more common than other psychiatric conditions. Substance use disorders were the third highest. In comparison with international studies, our results are slightly different from a Saudi Arabian study, whereby the most common diagnoses amongst sexual offenders referred for forensic assessment were substance abuse and addictive disorders, which accounted for 56% of their study population.^20^ Additionally, study results from the Netherlands^5^ reported that developmental disorders largely outnumbered other psychiatric disorders in terms of prevalence amongst offenders accused of sexual crimes. In our study, all diagnoses were obtained from the final reports compiled by the investigating psychiatrist(s) assigned to the cases. Minor diagnoses such as anxiety and depressive disorders were not found in this sample. A possible reason is that only more severe abnormal mental illnesses (as defined in the Criminal Procedure Act) meet the threshold criteria for observation in terms of Section 78 (accountability).^3^ Offenders with these minor mental illnesses are not likely to be referred for forensic observation as they are screened by the Director of Public Prosecution and are often referred for short court evaluations.

Alcohol and cannabis were the most used substances amongst our study population as comorbidities. This is not surprising as South Africans were found to use cannabis the most in comparison to other illegal substances, as reported by a 2012 national population-based survey.^21^ This result is comparable to international findings as evidenced by a study in the United States of America in which cannabis was found to be the most abused substance amongst sexual offenders.^10^ More than 10% of our study population also displayed patterns of multiple substance abuse comorbidly.

Regarding the constructs of competency to stand trial and criminal responsibility, it was found that 53.2% of the alleged offenders in our sample were fit to stand trial and 43.7% criminally responsible for their offences. Du Plessis et al.^22^ observed a comparable trend when 35% of offenders accused of rape and murder were found to be criminally responsible at the same study site. In our study, the issues of fitness to stand trial and criminal responsibility were found to be highly associated with mental illness, as 62.6% of mentally ill offenders accused of rape were not competent to stand trial, whereas 44.6% were pronounced not criminally responsible. Intellectual disability also had an impact on matters related to competency to stand trial and criminal responsibility. In comparison to a previous local study^14^ at the same study site, alleged offenders suffering from neurological conditions and comorbid intellectual disability were mostly not fit to stand trial nor responsible for their crimes, which included rape as the predominant offence.

### Limitations

A limitation of the study was that some files were incomplete with important data missing, or some documentation was not completed correctly. Documents such as the SAPS dockets, for example, were incomplete with regard to demographic information of alleged offenders of rape at the time of the crime, which resulted in only demographic information being available at the time of observation. Extended time periods from arrest to observation further contributed to the lack of accurate information at the time of committing rape offences.

## Conclusion

In this study, only males were found to have committed rape. These male offenders had a median age of 30 years, with ages ranging from 14 to 76 years. Most accused offenders had lower levels of education, placing almost half of the study population at a primary school level. A noteworthy section of the study population was unemployed and received government grants for disability or pension. The majority of offenders accused of rape were single.

Regarding clinical characteristics, schizophrenia spectrum and other psychotic disorders were the most prevalent diagnosis, followed by intellectual disability. Almost a third of the offenders accused of rape did not suffer from any form of mental illness. In addition to primary clinical diagnoses, alcohol and cannabis use disorders were the two most dominant comorbidities reported.

In terms of competency to stand trial, the multi-professional team found more than half of the accused offenders of rape to be competent after a 30-day forensic evaluation period. Most of this study sample were declared criminally responsible. Nearly 40% of offenders were competent to stand trial and criminally responsible for their actions. A third of the accused offenders were found not competent to stand trial and not criminally responsible.

This study provides valuable information on the sociodemographic, clinical and forensic characteristics of offenders accused of rape in the Free State, South Africa. These findings concerning the profile of offenders accused of rape are echoed to a large extent by other local studies conducted at the same study site.^4,13,14^

Although this study aims to contribute to international literature, it should be interpreted with care as far as generalisation of results is concerned as only offenders from the Free State, South Africa, were included in this study, consequently limiting the geographical area.

This study supports the link between low socio-economic status and limited education and the crime of rape. If criminal behaviour resulting in rape is to be reformed, it is suggested that prosocial sex behaviour programmes should be focused on at the primary school level in lower socio-economic sectors.
